# Systemic host inflammation induces stage-specific transcriptomic modification and slower maturation in malaria parasites

**DOI:** 10.1128/mbio.01129-23

**Published:** 2023-07-14

**Authors:** Lianne I. M. Lansink, Oliver P. Skinner, Jessica A. Engel, Hyun Jae Lee, Megan S. F. Soon, Cameron G. Williams, Arya SheelaNair, Clara P. S. Pernold, Pawat Laohamonthonkul, Jasmin Akter, Thomas Stoll, Michelle M. Hill, Arthur M. Talman, Andrew Russell, Mara Lawniczak, Xiaoxiao Jia, Brendon Chua, Dovile Anderson, Darren J. Creek, Miles P. Davenport, David S. Khoury, Ashraful Haque

**Affiliations:** 1 QIMR Berghofer Medical Research Institute, Herston, Brisbane, Queensland, Australia; 2 Department of Microbiology and Immunology, Peter Doherty Institute for Infection and Immunity, University of Melbourne, Parkville, Victoria, Australia; 3 School of Biomedical Sciences, Queensland University of Technology, Brisbane, Queensland, Australia; 4 Department of Biology, University of York, Wentworth Way, York, Yorkshire, United Kingdom; 5 Walter and Eliza Hall Institute of Medical Research, Parkville, Victoria, Australia; 6 Wellcome Sanger Institute, Wellcome Genome Campus, Hinxton, Cambridgeshire, United Kingdom; 7 MIVEGEC, University of Montpellier, IRD, CNRS, Montpellier, France; 8 Broad Institute of MIT and Harvard, Cambridge, Massachusetts, USA; 9 Monash Institute of Pharmaceutical Sciences, Monash University, Parkville, Victoria, Australia; 10 The Kirby Institute, University of New South Wales, Kensington, Sydney, New South Wales, Australia; Washington University in St. Louis School of Medicine, St. Louis, Missouri, USA; Washington University in St. Louis School of Medicine, St. Louis, Missouri, USA

**Keywords:** malaria, *Plasmodium*, parasite maturation, host inflammation, single-cell transcriptomics, metabolomics, host-parasite interaction

## Abstract

Maturation rates of malaria parasites within red blood cells (RBCs) can be influenced by host nutrient status and circadian rhythm; whether host inflammatory responses can also influence maturation remains less clear. Here, we observed that systemic host inflammation induced in mice by an innate immune stimulus, lipopolysaccharide (LPS), or by ongoing acute *Plasmodium* infection, slowed the progression of a single cohort of parasites from one generation of RBC to the next. Importantly, plasma from LPS-conditioned or acutely infected mice directly inhibited parasite maturation during in vitro culture, which was not rescued by supplementation, suggesting the emergence of inhibitory factors in plasma. Metabolomic assessments confirmed substantial alterations to the plasma of LPS-conditioned and acutely infected mice, and identified a small number of candidate inhibitory metabolites. Finally, we confirmed rapid parasite responses to systemic host inflammation *in vivo* using parasite scRNA-seq, noting broad impairment in transcriptional activity and translational capacity specifically in trophozoites but not rings or schizonts. Thus, we provide evidence that systemic host inflammation rapidly triggered transcriptional alterations in circulating blood-stage Plasmodium trophozoites and predict candidate inhibitory metabolites in the plasma that may impair parasite maturation *in vivo*.

**IMPORTANCE:**

Malaria parasites cyclically invade, multiply, and burst out of red blood cells. We found that a strong inflammatory response can cause changes to the composition of host plasma, which directly slows down parasite maturation. Thus, our work highlights a new mechanism that limits malaria parasite growth in the bloodstream.

## INTRODUCTION

Malaria, caused by infection with *Plasmodium* parasites, remains a significant global health problem, with ~200 million cases and ~400,000 deaths each year between 2015 and 2020 (1). *Plasmodium* parasites invade red blood cells (RBCs) and then mature, replicate, and burst out over ~24-, 48-, or 72-hour periods, depending on the infecting species. Given each parasite produces up to 32 daughter merozoites, parasite populations can increase dramatically *in vivo*. Malaria symptoms occur during this asexual, cyclical stage, with disease severity and risk of death during *Plasmodium falciparum* infection correlating positively with parasite biomass (2).

From a host perspective, slowing down parasite population growth constitutes an effective strategy for preventing or ameliorating severe disease. This can be achieved in multiple ways, for example, by killing parasites with artemisinin-based antimalarial drugs (3, 4) or blocking their transit between RBCs with parasite-specific antibodies (5). From a parasite perspective, there is theoretical benefit to adjusting the rate of progression through the asexual life cycle to avoid overwhelming a vulnerable host at least until transmission is guaranteed.

*In vitro* experiments support the idea that certain molecules can control asexual stage biology in *Plasmodium* parasites. For example, isoleucine starvation and lactate supplementation have been reported to slow blood-stage *P. falciparum* maturation *in vitro* (6 - 8). Lysophosphatidylcholine was reported to suppress cellular differentiation of asexual parasites into gametocytes (9). While causal links remain to be provided *in vivo*, the prevailing model is that many different types of metabolites could directly influence asexual stage *Plasmodium* parasites. In recent years, *in vivo* evidence from our group and others supports the idea that *Plasmodium* parasites can sense and rapidly respond to dynamic changes within the mammalian host. Mancio-Silva et al. demonstrated that *P. berghei* parasites up-regulated expression of ~180 genes within 6 hours of *in vivo* exposure to a calorie-restricted host—compared to control hosts fed *ad libitum* (10). Rijo-Ferreira et al*.* revealed altered periodicity of expression for ~1,000 *P*. *chabaudi* genes in hosts both harboring a genetically altered circadian rhythm and being housed without light-dark cycling (11). We showed the asexual life cycle was plastic for *P. berghei* or *P. yoelii* parasites (12), with the *P. berghei* parasite life cycle extended from ~24 to ~40 hours by exposure to a pre-existing malaria infection (12). Given this effect was lost in similarly infected immune-deficient *rag1^−/−^* mice, we surmised that host-mediated responses, perhaps systemic inflammation, had slowed maturation of parasites. Since parasitized RBCs (pRBCs) circulate in plasma, we hypothesized in this study that alterations to plasma constituents directly mediate adjustments to parasite maturation rate.

In addition to changes in the host environment, we also aimed to understand the parasite changes contributing to delayed development in the context of host inflammation. Cellular change in eukaryotic cells, including protozoan parasites, can be detected by transcriptomic analysis. Bulk RNA-seq and microarray studies of synchronized *Plasmodium* parasites have yielded stage-specific gene expression signatures that have been crucial for mapping biological processes accompanying asexual parasite maturation (13, 14). More recently, single-cell RNA-seq (scRNA-seq) has enabled examination of *Plasmodium* parasites without requirement to synchronize or otherwise fractionate parasites into morphologically similar subsets (15). These single-cell assessments have served to construct a “Malaria Cell Atlas” (MCA), which details transcriptomic changes as malaria parasites differentiate within mammalian and insect hosts (15). In these reports, the asexual stages of the *P. berghei* MCA were generated *in vitro* in the absence of host immune pressure. Hence, the possible effect of host inflammation on individual asexual parasites remains unknown.

Here, we employed *P. berghei* ANKA (*Pb*A) infection or innate immune stimulation of mice in conjunction with unsupervised metabolomic assessment of plasma, *in vitro* culturing methods, and droplet-based scRNA-seq of parasites to reveal that systemic host inflammation triggers changes to the host plasma environment, which is rapidly sensed and responded to by parasites residing in RBCs.

## MATERIALS AND METHODS

### Mice

C57BL/6J mice were purchased from the Animal Resource Centre (Perth, Australia). C57BL/6J.*rag1^−^/*^−^ mice were bred at QIMR Berghofer Medical Research Institute. All mice were female between 6 and 12 weeks of age and were maintained under conventional conditions. This study was carried out in strict accordance with guidelines from The National Health and Medical Research Council of Australia. All animal procedures and protocols were approved (A02-633M and A1503-601M) and monitored by the QIMR Berghofer Medical Research Institute Animal Ethics Committee.

### Generation of mice acutely infected with *Plasmodium*

*Plasmodium berghei* ANKA parasites were sourced and used as previously reported (12, 16, 17). *Pb*A parasites were used after defrosting cryopreserved infected blood and a single *in vivo* passage in C57BL/6J mice. RBCs were collected from passage mice by cardiac puncture and used to infect with 10^5^ pRBCs via lateral tail vein injection. On day 5 post-infection, these mice were categorized as being acutely infected, confirmed by flow cytometric assessment of parasitemia.

### Adoptive transfer of fluorescently labeled parasitized red blood cells

Adoptive transfer of fluorescently labeled RBCs was performed as previously described (5, 12). Briefly, RBCs were collected from infected mice by cardiac puncture, washed twice in Ca^2+^- and Mg^2+^-free PBS, and stained with viable cell dye CellTrace Far Red (CTFR; Thermo Fisher, Waltham, Massachusetts, USA), according to manufacturer’s instructions. CTFR-labeled RBCs were resuspended in 2 mL of Roswell Park Memorial Institute (RPMI) media per donor mouse and injected in 200 µL volumes to recipient mice via lateral tail vein injection using 26G needles.

### TLR agonist treatment

Mice were treated with saline (0.9%; Baxter, Deerfield, Illinois, USA), lipopolysaccharide (LPS; 0.75 mg/mL) (Sigma-Aldrich, St. Louis, Missouri, USA), CpG 1826 (0.25 mg/mL) (Sigma-Aldrich), or polyriboinosinic:polyribocytidylic acid (Poly I:C; 2 mg/mL) (InvivoGen, San Diego, California, USA) via intraperitoneal injection (200 µL per mouse) using 26G needles, 2 hours prior to adoptive transfer of CFTR-labeled RBCs.

### Flow cytometry of pRBC

Flow cytometric analysis of pRBCs in peripheral blood was performed as previously described (12). One to two drops (approximately 10–20 µL) of peripheral blood were collected by tail vein bleed and diluted in RPMI medium containing 5 U/mL heparin sulfate. Diluted blood was stained with Hoechst33342 (10 µg/mL) (Sigma-Aldrich) and Syto84 (5 µm) (Life Technologies, Carlsbad, California, USA) at room temperature in the dark for 30 minutes. Staining was quenched with 10× initial volume of ice-cold RPMI medium, and samples acquired using an LSR II Fortessa Analyzer (BD Biosciences, Franklin Lakes, New Jersey, USA) and analyzed using FlowJo Software version 10.7 (Treestar, Ashland, Oregon, USA).

### Cytokine analysis

Serum or plasma cytokine levels were assessed using a murine soluble protein Cytometric Bead Array Flex Set System (BD Biosciences), as per manufacturer’s instructions. Data were acquired on a BD LSR II Fortessa Analyzer (BD Biosciences) and analyzed using FCAP Array Software version 3.0 (BD Biosciences).

### Plasma metabolomics

Two independent experiments were conducted, each with six mice per treatment group. Ice-cold butanol/methanol (1:1) containing 50 µg/mL antioxidant 2,6-di-tert-butyl-4-methylphenol (BHT) was added to each plasma sample, as well as a pooled quality control (QC) sample at 10× volume. Samples were vortexed for 10 seconds, then snap frozen on dry ice. Thawed samples were sonicated for 15 minutes on ice, stored for 2 hours at −30°C, and then centrifuged for 15 minutes at 16,000 × *g* (4°C). Supernatant was collected, aliquoted, and dried down using a vacuum concentrator and stored at −80°C until liquid chromatography-coupled mass spectrometry (LC/MS) analysis.

Untargeted metabolomics was performed as published previously (18) with slight modifications, using a 1290 Infinity II UHPLC coupled to a 6545 QTOF Mass Spectrometer via Dual AJS ESI source (Agilent, Santa Clara, California, USA) and MassHunter Data Acquisition Software (version 10.1). Full-scan MS data (m/z 50–1,700) was acquired at a scan rate of 2.5 spectra/s with the following source conditions: gas temperature 250°C, gas flow 13 L/min, sheath gas temperature and flow at 400°C and 12 L/min, respectively, nebulizer 30 psi, fragmentor 135, capillary voltage at +4,500 V and −4,000 V, nozzle voltage was zero. Metabolite separation was performed on a Zorbax HILIC Plus RRHD (95 Å, 1.8 µm, 2.1 × 100 mm, Agilent) analytical column connected to a 3 × 5 mm Zorbax HILIC Plus UHPLC guard column. The autosampler and column temperature were set to 4°C and 40°C, respectively. In positive and negative mode, eluent A was 10 mM ammonium acetate in acetonitrile/milliQ water (95:5, vol/vol) and eluent B was 10 mM ammonium acetate in acetonitrile/milliQ water (50:50, vol/vol). The following gradient was used for both modes: 0 minute (1% eluent B), 3.5 minutes (50% B), 5.5 minutes (99% B), 6.5 minutes (99% B), 6.7 minutes (1% B), 12 minutes (1% B). Flow rate was set to 0.5 mL/min.

For LC/MS analysis, polar compounds were resuspended by addition of milliQ water, thoroughly vortexing, and incubating for 10 minutes on ice. Following centrifugation, supernatant was transferred to a new tube and diluted with chilled acetonitrile containing 1 µg/mL Val-Tyr-Val internal standard to a final concentration of 80% acetonitrile. Injection volumes were 2 µL for positive and 3 µL for negative mode. Samples were run in a randomized order.

Positive and negative mode data were analyzed separately using MassHunter Profinder (version 10 SP1, Agilent) recursive feature extraction method, employing default settings with minor adjustments: peak extraction was restricted to retention time (Rt) range 0–6.5 minutes, compound binning and alignment tolerances were set to 1% + 0.3 minutes for Rt and 20 ppm +2 mDa for mass, integrator Agile 2 was used for peak integration, peak filters were set to at least 2,500 counts, and features must have satisfied filter conditions in at least 75% of files in at least one sample group. Feature peak area was exported, and data cleaning was performed using an in-house R script compiled of the following steps. Features were deleted if they: had a mean QC/tube blank area ratio of <10, were absent across all QC samples, and had duplicates present. In addition, samples with a total ion current (TIC) scaling factor more than 50% above or below the median TIC were removed. Differential expression analysis and principal component analysis (PCA) were conducted using MetaboAnalystR 2.0 (19), including additional data pre-processing steps: features with >50% missing values were removed, missing data values were imputed using k-nearest neighbor (kNN), sample normalization was performed by reference sample (probabilistic quotient normalization), and a log transformation was applied.

For metabolite identification, LC/MS was conducted using an in-house reference standards library from Monash Proteomics and Metabolomics Platform (MPMP). Briefly, seven different mixtures of authentic standards mixed by separating isobaric mass compounds were acquired together with the batch of samples. Approximately, 350 standards are routinely detected. Retention times for the MPMP reference standard library were extracted using the targeted peak detection module in MZmine 2.53 (20). Peak extraction was performed using 3 ppm mass error and 1 minute retention times windows. Samples were acquired on a Q-Exactive Orbitrap Mass Spectrometer (Thermo Fisher) coupled with high-performance liquid chromatography system Dionex Ultimate 3000 RS (Thermo Fisher). Chromatographic separation was performed on a ZIC-pHILIC column (5 µm, polymeric, 150 × 4.6 mm, SeQuant; Merck, Rahway, New Jersey, USA). The mobile phase (A) was 20 mM ammonium carbonate and (B) acetonitrile. The gradient program started at 80% B and was reduced to 50% B over 15 minutes, then reduced from 50% B to 5% B over 3 minutes, followed by wash with 5% B for another 3 minutes, and finally 8 minutes re-equilibration with 80% B. The flow rate was 0.3 mL/min, and column compartment temperature was 40°C. The total run time was 32 minutes with an injection sample volume of 10 µL. Mass spectrometer was operated in full scan mode with positive and negative polarity switching at 35 k resolution at 200 m/z with detection range of 85–1,275 m/z in full scan mode. Electro-spray ionization source (HESI) was set to 4.0 kV voltage for positive mode and 3.5 kV for negative mode, sheath gas was set to 50 and aux gas to 20, and sweep gas to 2 arbitrary units, capillary temperature 300°C, probe heater temperature 120°C, and S-lens 50. Data were processed with the IDEOM software (21) using default parameters. Metabolite identification (MSI level 1) was based on accurate mass (<2 ppm) and retention time of authentic standards analyzed in the same batch. Putative annotation of remaining features (MSI level 2) was based on accurate mass (<2 ppm) and predicted retention time (22) (trained on the 349 authentic standards) from the IDEOM metabolite database. Peak intensity data were log10 transformed and autoscaled using MetaboAnalyst 5.0 (23). The same software package was used to generate principal component score plots, hierarchical clustering heatmaps, and pattern hunter analysis. Each number in the pattern represented the expected expression level in the corresponding treatment group. Pearson correlation coefficients were then calculated for each metabolite based on fit to the pre-defined pattern.

### *In vitro Pb*A maturation assay

*Pb*A-infected blood was added to culture medium (RPMI, 5 U/mL heparin sulfate) at 2.5% hematocrit, containing 10% vol mouse plasma. Samples were cultured in 5% O_2_, 5% CO_2_, and 90% N_2_ at 37°C for 22 hours.

### Droplet-based scRNA-seq of parasitized RBC

Peripheral blood (five to six drops) was collected into 1 mL cold RPMI medium containing 5 U/mL heparin by tail vein bleed. Diluted blood (50 µL) was further diluted in 2 mL cold 1% BSA/PBS, and RBCs isolated away from leukocytes by cell sorting. RBCs, containing pRBCs, were loaded such that ~5,000 pRBCs were loaded per channel onto a Chromium Controller (10x Genomics, Pleasanton, California, USA) for generation of gel-bead-in-emulsions. Sequencing libraries were prepared using Single Cell 3' Reagent Kits version 3.1 for main experiment or version 2 for repeat experiment (10x Genomics) and sequenced on a NextSeq550 (Illumina, San Diego, California, USA) using paired-end 150- base pair reads.

### scRNA-seq data quality control and analysis

FASTQ files were processed using Cell Ranger version 2.1.0 (10x Genomics) for the reference data set and version 3.0.2 for the study of impaired maturation with combined genomes of mouse (mm10—Genome Reference Consortium) and *Pb*A [*PlasmoDB* release 39 (13)] as a reference (24). For quality control, RBCs were first classified as containing only parasite signal, mouse, or both. Using Cell Ranger, all genes of mouse origin were filtered out. Seurat version 3.2.2 “SCTransform” function was used for normalization, finding of variable features, and scaling of data (25). Uniform manifold approximation and projection (UMAP) for dimension reduction (26) coordinates were generated using Seurat’s “RunUMAP” function based on 10–15 principal components (PCs). Differential gene expression analysis was performed with Seurat’s “FindMarkers” function using Wilcoxon-rank sum test, with all parameters kept at default except min.pct = 0.25. Integrated dimensionality reduction with the malaria cell atlas data sets was performed using single-cell variational inference (scVI) version 0.7.1 (27), with all parameters kept at default except dispersion parameter set as “gene-batch.” Each data set was identified as separate batch. Computed latent variables were used as input to generate UMAP using Seurat’s RunUMAP function. To infer parasite life stage from scRNA-seq data, *PlasmoDB* was used to extract *Pb*A genes from bulk RNA-seq data (13), up-regulated at least twofold for each asexual blood stage compared to the highest average gene expression value in the remaining two life stages, resulting in lists of 291 genes for to ring, 581 genes for trophozoites, and 754 genes for schizont stages. Mean expression of all genes in these genes signature lists was calculated for each cell and visualized using ggplot2 package. The scRNA-seq datasets generated during this study were deposited at ArrayExpress.

### Statistics

Statistical analyses were performed using Prism 7 version 7.0c (GraphPad Software, San Diego, California, USA) or R version 3.6.3, the latter was used only for statistical analysis, one-way analysis of variance (ANOVA), of transcript numbers from scRNA-seq data. Time course graphs depict either individual data points or mean ± standard deviation. Anderson-Darling or Shapiro-Wilk normality tests were used to test the appropriateness of subsequent parametric statistical testing. Time course experiments were analyzed using either two-way ANOVA multiple comparisons test, multiple comparisons test with a factor for timepoint and a factor for treatment group (either Tukey or Dunn-Šidák corrected), or mixed-effect analysis as described in figure legend. Other analyses include one-way ANOVA and *t*-test as described in figure legend. A *P* value of 0.05 was considered significant. *P* values are shown as *P < 0.05, **P < 0.01, ***P < 0.001, ****P < 0.0001.

## RESULTS

### Systemic host inflammation impairs maturation of blood-stage parasites *in vivo*

**FIG 1:**
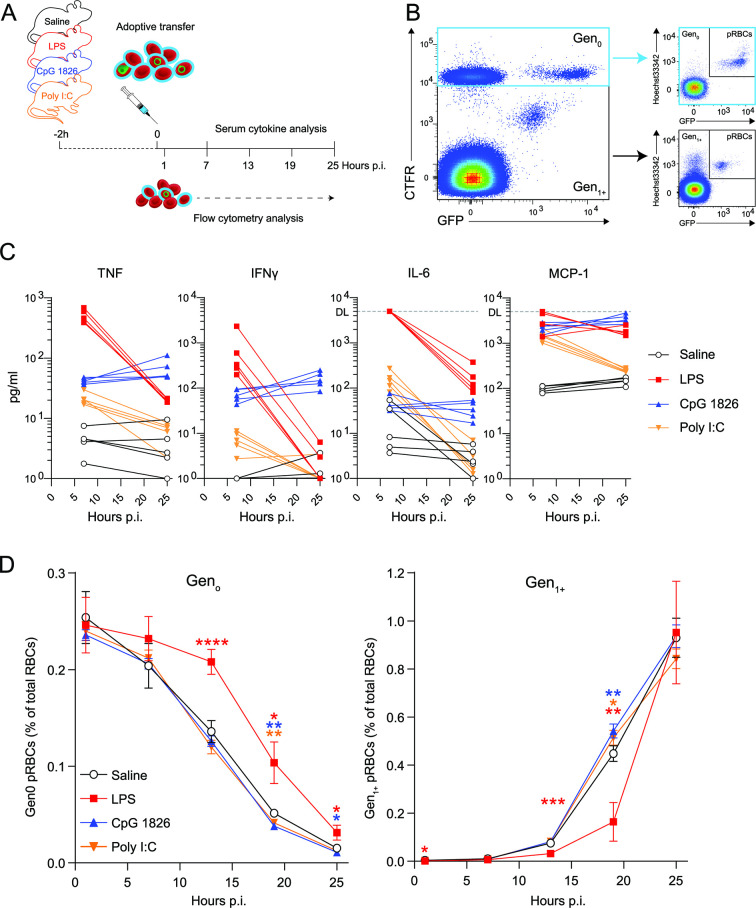
LPS-induced systemic inflammation impairs blood-stage parasites *in vivo*. (**A**) Schematic of CTFR-labeled RBCs (~2%–5% containing *Pb*A-eGFP parasites) transferred into recipient mice (*n* = 5/group), 2 hours after treatment with LPS (150 µg), CpG 1826 (50 µg) or Poly I:C (400 µg) or control saline, with peripheral blood assessed by flow cytometry at timepoints indicated. (**B**) Representative flow cytometry gating for Gen_0_ (CTFR^+^) and Gen_1+_ (CTFR^−^) RBCs and assessment of parasites via Hoechst33342 and GFP expression. (**C**) Serum cytokine levels tracked in individual mice at 7 and 25 hours post-infection—dotted line: assay detection limit (DL). (**D**) Enumeration of Gen_0_ and Gen_1+_ pRBCs over time. Data presented as mean ± SD. Statistical significance tested relative to saline controls. Data representative of two independent experiments. Statistical analysis: two-way ANOVA with a factor for timepoint and for treatment group. Testing for a treatment group effect in Gen_0_ [*P* < 0.0001, *F* = 20.46, degrees of freedom (df) = 3] and Gen_1+_ (*P* = 0.0087, *F* = 5.492, df = 3). *P < 0.05, **P < 0.01, ***P < 0.001, ****P < 0.0001 (Dunnett test for multiple comparisons).

In this study, we used our established method of RBC adoptive transfer into mice to study maturation of a single cohort of blood-stage *Plasmodium* parasites (3 - 12 - 28 - 28). In this method, fluorescently labeled RBCs containing *Pb*A-eGFP [defined as Generation 0 (Gen_0_)] were transferred into recipient mice, with flow cytometric enumeration and life staging in peripheral blood conducted every 4–6 hours. To determine if systemic host inflammation alone could impair parasite maturation *in vivo*, mice were pre-treated with Toll-like Receptor (TLR) 4, TLR9 or TLR3 agonists (LPS, CpG or Poly I:C, respectively), or control saline, and then injected with a cohort of CTFR-labeled RBCs containing *Pb*A-eGFP (Fig. 1A). After parasites matured within Gen_0_, they burst out of CFTR^+^ RBCs (which constituted a minority of all RBCs), with the majority of resulting merozoites infecting CFTR^−^ RBCs, defined as Generation 1 (Gen_1+_) (Fig. 1B). This technique permitted examination of parasite transit from one generation of RBC to the next *in vivo*.

First, we confirmed as expected that all TLR agonists stimulated systemic cytokine production, as markers of systemic inflammation, by 9 hours post-treatment, with significant increases in tumour necrosis factor (TNF), interferon γ (IFNγ), and interleukin (IL)-6, and chemokine, MCP-1 (also known as CCL2) (Fig. 1C). At the doses employed, quantitative and qualitative differences were evident between agonists (Fig. 1C). LPS triggered the largest systemic cytokine response by 9 hours post-treatment, CpG the most prolonged up-regulation of TNF and IFNγ, while Poly I:C elicited the mildest response. We next examined the fate of Gen_0_ and Gen_1+_ parasites by flow cytometry. As expected (12, 28) in saline-treated control mice, the majority of Gen_0_ parasites were gradually lost over 25 hours, through rupture and transition to Gen_1+_, and via host clearance, as previously implicated (3). We observed no effect of CpG or Poly I:C treatment on the dynamics of Gen_0_ or Gen_1+_ parasites (Fig. 1D). In contrast, LPS treatment caused parasites to persist in Gen_0_, which was also associated with a delay in the emergence of Gen_1+_ parasites (Fig. 1D). These data revealed that LPS, but not CpG or Poly I:C at the doses employed, slowed the rate at which parasites transitioned from one RBC to the next *in vivo*. These data provided proof of concept that inflammation induced by LPS and parasitic infection could delay maturation of *Plasmodium* parasites within RBCs.

### Host plasma conditioned by systemic inflammation directly impairs parasite maturation

**FIG 2:**
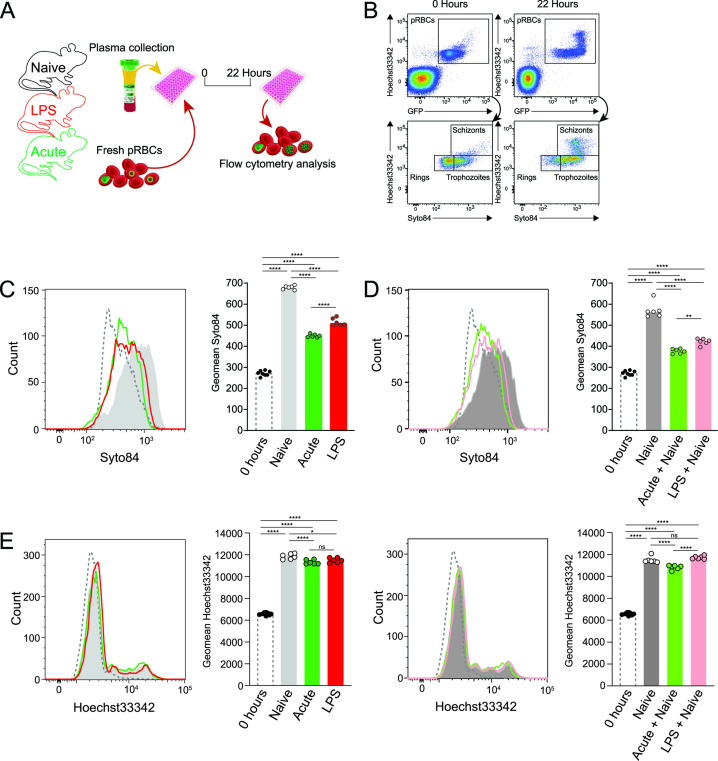
Plasma altered by systemic inflammation directly inhibits parasite maturation. (**A**) Schematic showing pRBCs from a passage mouse cultured *in vitro* for 22 hours, in media supplemented with pooled plasma from LPS-conditioned, acutely infected or control mice (*n* = 6/group), followed by flow cytometric assessment of maturation. (**B**) Representative flow cytometry gating on unconditioned wild-type blood to identify pRBCs using Hoechst33342 staining and GFP expression, and life stages using Hoechst33342/Syto84 staining at 22 hours p.i. compared to 0 hours. (**C**) Representative histogram of Syto84 expression in non-schizont parasites after culture—10% vol/vol plasma. Bar graph shows geometric mean Syto84 staining of technical replicates, data representative of two independent experiments. (**D**) Representative histogram of Syto84 expression in non-schizont parasites after culture with plasma from LPS-conditioned or acutely infected mice supplemented with equal volumes naïve plasma—10% + 10% vol/vol plasma in all groups. Bar graph shows geometric mean of Syto84 staining in six technical replicates, data representative of two independent experiments. (**E**) Representative histogram of Hoechst33342 staining in all pRBCs prior to and after culture as in (C) and (D), with data representative of six technical replicates in two independent experiments. Data shown are the median with statistical test used one-way ANOVA with Tukey’s multiple comparisons test. *P < 0.05, **P < 0.01, ****P < 0.0001.

We next hypothesized that delayed maturation was mediated by exposure of parasites to an altered plasma environment as they circulated in the bloodstream. To test this, we cultured pRBCs in media supplemented with plasma from naïve, LPS-conditioned, or acutely infected hosts (Fig. 2A). We employed a previously reported *in vitro* maturation assay (10) and examined life-stage progression of *P. berghei* ANKA parasites by flow cytometry (Fig. 2B). Although ring/early trophozoites matured effectively in the presence of plasma from naïve mice, this was significantly impaired in the presence of plasma from mice either acutely infected or LPS-conditioned (Fig. 2C). Moreover, the defect in maturation was not rescued by supplementation with naïve plasma (Fig. 2D). In addition, it was also noted that schizont development was less disrupted (Fig. 2E). Together, these data demonstrated that host plasma from LPS-conditioned or acutely infected mice directly delayed maturation of ring and early trophozoite stages, with little effect on mature trophozoites progressing to schizont stages. Moreover, attempted supplementation suggested the presence of inhibitory factors in plasma as a result of acute infection or LPS conditioning.

### Systemic inflammation alters the metabolomic composition of host plasma

**FIG 3:**
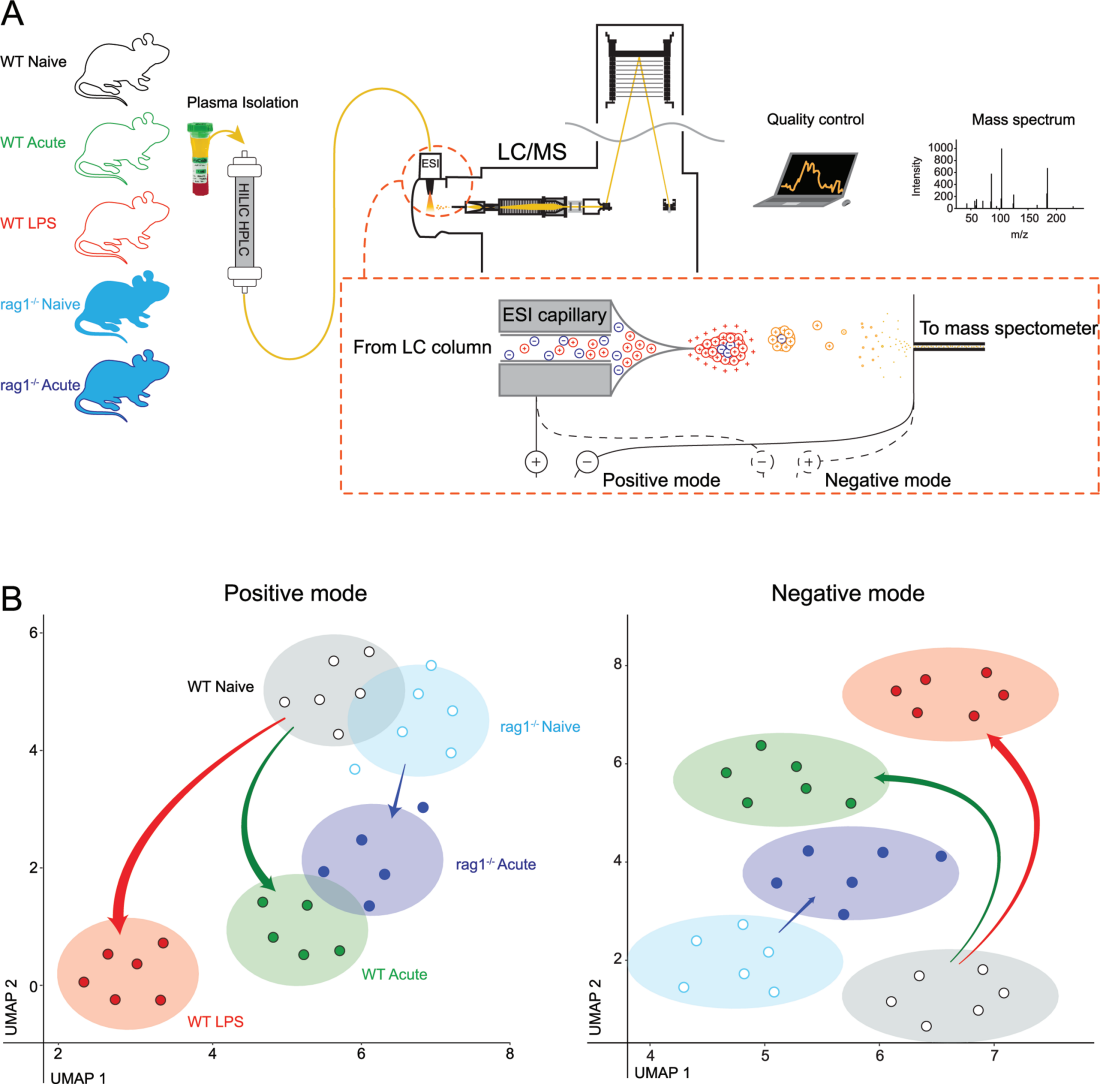
Systemic inflammation alters the metabolomic profiles of circulating plasma *in vivo*. (**A**) Schematic showing plasma samples (*n* = 6 per group) separated on a hydrophilic interaction liquid chromatography (HILIC) column, followed by ionization and mass spectrometry. (**B**) UMAP dimensionality reduction representation of untargeted LC/MS data obtained using positive or negative electrospray ionization (ESI). Dots represent plasma metabolomes from individual mice, shaded ellipses depict centroid, and 95% confidence intervals for each group. Arrow width indicates Euclidean distance between centroids of groups. Independent experimental repeat shown in Fig. S1.

We next determined whether LPS conditioning and acute *Plasmodium* infection had altered plasma composition via metabolomic assessments. We included as negative controls plasma from acutely infected and naïve *rag1^−/−^* mice since parasite maturation is not substantially delayed in these hosts (12). Untargeted metabolomics using LC/MS with either positive or negative ionization was conducted on plasma samples from individual mice (Fig. 3A). Abundance of metabolite features for each sample was analyzed by PCA and 2D-UMAP in which proximity of samples indicated metabolomic similarity (Fig. 3B). Plasma samples from mice in the same experimental group clustered together, revealing as expected similar metabolomes among similarly treated mice (Fig. 3B). Next, consistent with our hypothesis, plasma metabolomes from LPS-conditioned and acutely infected wild-type mice did not overlap with naïve wild-type controls or each other (Fig. 3B, and an independent experimental repeat in Fig. S1). Although plasma metabolomes were altered by acute malaria in *rag1^−/−^* mice, this occurred to a lesser degree than in wild-type mice (Fig. 3B). Thus, LPS conditioning and acute infection had both altered plasma compositions.

### Plasma metabolomics identifies potential *in vivo* inhibitory factors of parasite maturation

**FIG 4:**
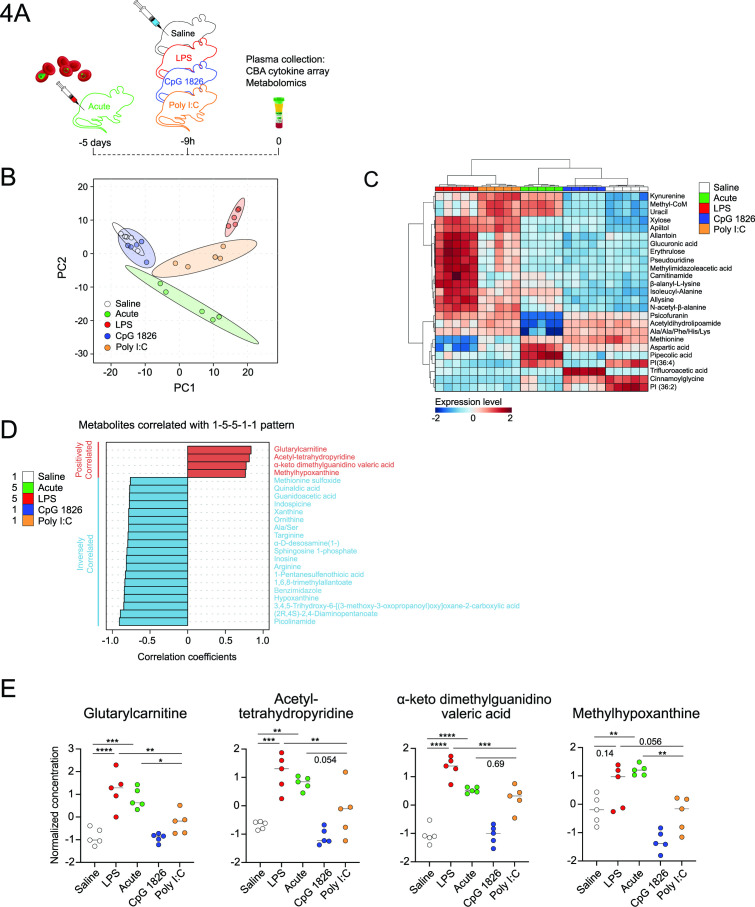
Plasma metabolomics identifies potential inhibitory factors of parasite maturation during systemically inflamed mice. (**A**) Schematic of experimental plan for assessing plasma metabolome in 5 days *Pb*A post-infected (acute) and 9 hours LPS, Poly I:C and CpG post-treated or saline control mice (*n* = 5 per group). Plasma collected from mice at the same time for CBA cytokine array or untargeted metabolomics. (**B**) Principal component plots of PC1 versus PC2 of the plasma metabolomics data set. Dots represent plasma metabolome from individual mice per treatment group. Ellipses around data points for each group represent the 95% confidence regions. (**C**) Hierarchical clustering heatmap showing the top 25 altered metabolites. (**D**) Pearson correlation coefficients were calculated to identify metabolites that correlate with the pattern 1-5-5-1-1 (with low expression as 1 to high expression as 5) in the sequence Saline, LPS, acute, Poly I:C, and CpG. Metabolites shown are those with correlation coefficients above 0.75 or below −0.75 that are statistically significant (false discovery rate [FDR] <0.05). (**E**) Dot plots showing the normalized concentration for the four positively correlated metabolites. Data shown are the median with statistical test used one-way ANOVA with Tukey’s multiple comparisons test. *P < 0.05, **P < 0.01, ***P < 0.001, ****P < 0.0001.

To search for potential inhibitory factors within the plasma of LPS-conditioned and acutely infected mice, we employed LC/MS metabolomics using high-resolution mass spectrometry with a reference library of 349 molecular standards. We included as negative controls the plasma from mice in which parasite maturation had been unaffected—saline-treated, CpG-conditioned, and Poly I:C-conditioned mice (Fig. 4A). First, we confirmed that acute infection or LPS, CpG, and Poly I:C conditioning had triggered expected systemic inflammation, as assessed by systemic pro-inflammatory cytokines (Fig. S2). Next, high-resolution LC/MS putatively identified and measured the relative abundance of 429 metabolites in the plasma samples (Fig. 4B). As with initial LC/MS conducted without standards (Fig. 3B), plasma from LPS-conditioned and acutely infected mice exhibited metabolite profiles distinct from saline controls as well as being distinct from each other. Of note, plasma from CpG-conditioned mice showed minimal alteration in metabolites relative to saline-treated controls (Fig. 4B). Hierarchical clustering heatmap analysis of the top 25 differentially abundant metabolites emphasized substantial differences between groups (Fig. 4C).

We next hypothesized the existence of common inhibitory factors that would be elevated in the plasma of both LPS-conditioned and acutely infected mice relative to the other three groups. To search for these, a “pattern hunter” analysis was performed, which revealed 89 statistically significant (FDR < 0.05) metabolites that positively correlated and 97 that inversely correlated with this pattern. Of metabolites positively correlating with this pattern, four exhibited correlation coefficients >0.75 (Fig. 4D). Of these, the metabolites putatively annotated as acetyl-tetrahydropyridine and alpha-keto dimethylguanidino valeric acid were also modestly elevated in Poly I:C-conditioned mice, reducing our interest in these (Fig. 4E). In contrast, glutarylcarnitine and methylhypoxanthine were elevated only in the treatments associated with impaired parasite maturation (Fig. 4E), suggesting these as potential common inhibitors of parasite maturation *in vivo*. The identification of glutarylcarnitine was confirmed by comparison of accurate mass and retention time with an authentic standard. Methylhypoxanthine was identified based on accurate mass and predicted retention time, as we were unable to obtain authentic standards to confirm its isomeric conformation. Among the 19 inversely correlated metabolites (with correlation coefficients less than −0.75), the metabolite putatively annotated as picolinamide was the most inversely correlated metabolite (Fig. 4D). We also noted inverse correlations for inosine, hypoxanthine, and xanthine, which are essential host-derived substrates acquired by *Plasmodium* for critical production of nucleic acids, adenosine, and guanine di- and tri-phosphates. Thus, plasma from LPS-conditioned and acutely infected mice harbored elevated levels of certain metabolites, which are therefore candidate inhibitory factors for parasite maturation *in vivo*.

### Droplet-based scRNA-seq maps asexual life cycle progression of *P. berghei* ANKA parasites *in vivo*

**FIG 5:**
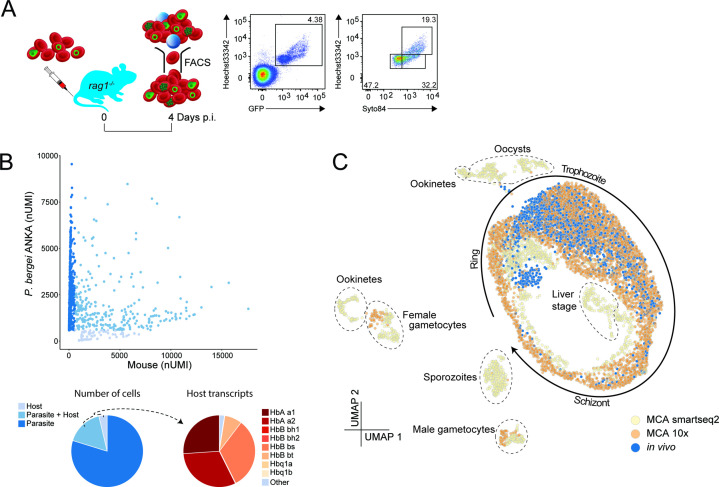
Droplet-based scRNA-seq of parasites directly *ex vivo*. (**A**) Schematic for assessing droplet-based scRNA-seq of blood-stage parasites directly *ex vivo*. Blood from *Pb*A-eGFP-infected *rag1^−/−^* mice was pooled (~4.4% parasitemia, spectrum of life stages as shown in fluorescence-activated cell sorting [FACS] plots) prior to FACS sorting of RBCs and exclusion of leukocytes via forward scatter/side scatter (FSC/SSC) profiles. RBCs were loaded onto a Chromium Controller for transcriptomic analysis. (**B**) Number of Unique Molecular Identifier (nUMI) transcripts mapping to mouse and parasite genomes for each cell. Pie charts show proportion of RBCs classified as containing transcripts from parasites only, host only, or a mixture of the two (left), and for host transcripts, the proportions encoding hemoglobin genes (right). (**C**) UMAP representation of scVI-integrated data of direct *ex vivo* blood stages from this study with MCA 10x and SMART-seq2 data.

To determine if parasites responded directly to altered plasma environments, we opted to analyze pRBCs *ex vivo* via transcriptomics. Asynchronicity of *P. berghei* ANKA parasites *in vivo*, and possible differential effects on ring, trophozoite, and schizont stages, necessitated a single-cell RNA-seq approach. Previous scRNA-seq work, presented in the Malaria Cell Atlas (15), largely examined *P. berghei* after *in vitro* culture, where host inflammatory effects and host RNA, particularly from recently invaded reticulocytes, would be absent. Therefore, to determine feasibility of analyzing individual parasite transcriptomes directly *ex vivo* and to compare these data to the MCA*,* we first studied pRBCs from infected *rag1^−/−^* mice (Fig. 5A) for easier detection of all asexual life stages including mature trophozoites and schizonts (29) and because host inflammatory effects would be reduced. After flow cytometric exclusion of white blood cells from whole blood, total RBCs, including those parasitized, were processed for droplet-based 3′ scRNA-seq (Fig. 5A). Dual mapping of scRNA-seq data to the *Plasmodium* and mouse genomes revealed the majority (79.8%) of RBCs with detectable mRNA solely contained parasite-derived transcripts, while 3.7% contained host RNA alone (almost exclusively hemoglobin-encoding mRNA), or a mixture of hemoglobin and parasite mRNA (Fig. 5B). These data suggested that direct *ex vivo* examination of parasites was not impeded by the presence of host RNA. To compare the quality of our direct *ex vivo* data to the MCA, which itself was generated by two different methods (SMART-seq2 and 10×), we performed neural network-based integration of these three data sets using single-cell Variational Inference (scVI) (27), as described before (30, 31), which highlights commonalities between data sets and removes technical batch effects. scVI-based integration revealed, as expected, no cross over between our *ex vivo* asexual stage data and the multiple sexual, liver and mosquito-stage parasites (Fig. 5C). Instead, our data mapped closely with the asexual life cycle stages of the MCA, and given the proportion of ring, trophozoite, and schizont stages present in our sample by flow cytometric analysis (Fig. 5A), we noted the majority of direct *ex vivo* transcriptomes mapped onto MCA zones associated with ring and trophozoite stages, with a minority occupying the more mature schizont stages (Fig. 5C). These data confirmed the feasibility of examining individual blood-stage parasite transcriptomes directly *ex vivo*, facilitating the screening of heterogeneous parasite populations after exposure to altered host plasma.

### Systemic host inflammation down-regulates transcriptional and translational activity in trophozoites

**FIG 6:**
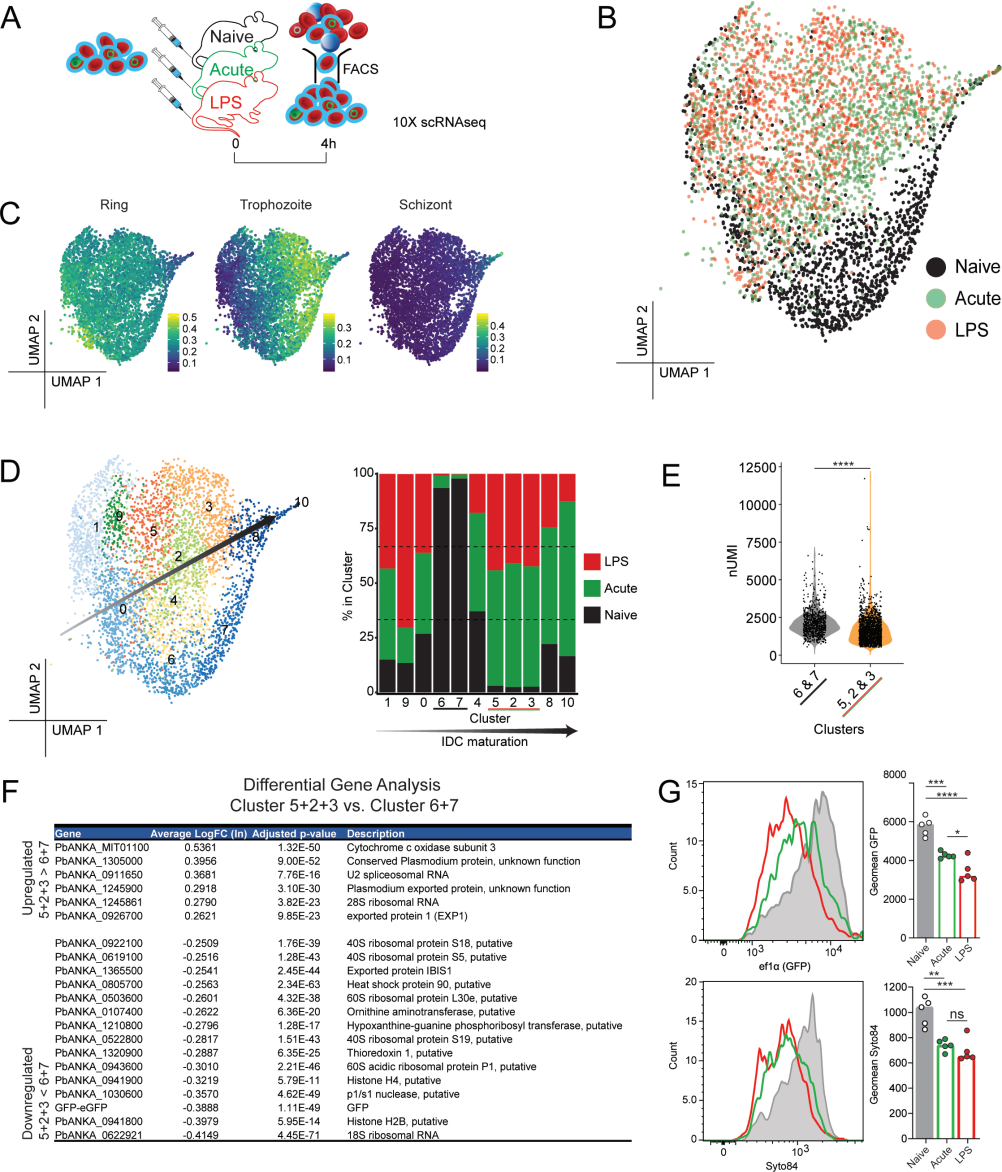
Rapid transcriptomic change in trophozoites exposed to systemic host inflammation *in vivo*. (**A**) Schematic showing CFTR^+^ RBCs (containing 4.8% *Pb*A-eGFP parasites) transferred into LPS-conditioned, acutely infected, or control mice (*n* = 5/group), recovered by cell sorting after 4 hours, and immediately loaded onto a Chromium Controller for scRNA-seq analysis. (**B**) 2D UMAP after PCA of post-QC individual parasite transcriptomes after 4 hours *in vivo* exposure to host-inflammation. (**C**) Expression of ring, trophozoite, and schizont gene signatures across the UMAP embedding taken from *PlasmoDB* (https://plasmodb.org/plasmo/app). (**D**) Relative contribution of parasites from each group within each of 11 clusters, defined by unsupervised clustering on transcriptomic similarity; arrow indicates inferred rough directionality of maturation from ring to trophozoite to schizonts within the pooled data. (**E**) Number of Unique Molecular Identifiers (nUMI) per cell for pooled clusters 6 + 7 and 5 + 2 + 3. (**F**) Differentially expressed genes between pooled clusters 5 + 2 + 3 versus pooled clusters 6 + 7 ranked by average Log_n_FoldChange. (**G**) Representative histograms of GFP expression driven off the elongation factor 1α promoter (top) and Syto84 staining (bottom) in non-schizonts 13 hours post-transfer; bar graphs show geometric mean GFP and Syto84 in individual mice (*n* = 5/group). Statistical analyses performed *t*-test for panel E and one-way ANOVA for G. (**E**) Testing for a grouped cluster effect (*P* < 0.0001, *t* = 160.9, df = 1). (**G**) Testing for a treatment group effect in GFP (*P* < 0.0001, *F* = 34.65, df = 2) and Syto84 (*P* = 0.0002, *F* = 18.35, df = 2). *P < 0.05, **P < 0.01, ***P < 0.001, ****P < 0.0001 (Tukey’s test for multiple comparisons).

To assess parasite transcriptomes directly *ex vivo*, we first reduced the time needed to FACS-sort transferred CFTR^+^ RBCs from peripheral blood of LPS-conditioned, acutely infected, or naïve hosts (Fig. 6A). Our published RBC adoptive transfer method (5, 12, 28) results in ~2%–3% of all RBCs in recipients being CFTR^+^ (Fig. S3A), a frequency incompatible with timely FACS-sorting within ~20 minutes. So, we first confirmed that increasing the number of CFTR^+^ RBCs injected per mouse by approximately fivefold elicited the same impaired maturation phenotype for Gen_0_ parasites as previously observed in acutely infected mice (Fig. S3B).

Next, we adoptively transferred this higher number of CFTR^+^ RBCs (~4% parasitized) equally among three different groups of conditioned hosts—LPS-conditioned, acutely infected, or control naïve. Given evidence of altered phenotypes in asexual parasites *in vivo* by 6 hours post-exposure (10, 12), we recovered CFTR^+^ RBCs after 4 hours to capture early transcriptional changes in Gen_0_ parasites. In parallel, we also confirmed at subsequent timepoints, 13 and 25 hours post-transfer, that LPS conditioning and acute infection had indeed caused Gen_0_ parasites to persist (Fig. S4A and B). Total CFTR^+^ RBCs recovered at 4 hours from each of five mice per conditioned host group were pooled in roughly equal proportions and immediately loaded for droplet-based scRNA-seq (Fig. 6A; Fig. S5A), with the objective of capturing ~3,000 parasite transcriptomes per condition.

After mapping to the *P. berghei* ANKA genome, and removal of low-quality pRBCs and uninfected RBCs, we recovered a total of 4,772 parasite transcriptomes across all three conditions (1,500 for LPS, 1,970 for acute-infection, and 1,302 for naïve). The UMAP revealed a heterogeneous spectrum of parasite transcriptomes in naïve mice (Fig. 6B; Fig. S5B). Strikingly, many transcriptomes from LPS-conditioned or acutely infected mice appeared to occupy the same unique areas of the UMAP away from naïve transcriptomes (Fig. 6B; Fig. S5B). Using publicly available gene signatures for ring, trophozoite, and schizont stages from *PlasmoDB*, we inferred ring stages dominated a minor portion on the left of the UMAP, with schizonts located toward an extreme right point of the data (Fig. 6C; Fig. S5C). We also validated *PlasmoDB*-based life-stage annotations by transposing these clusters onto an scVI-derived integrated map of all our data sets alongside the 10x Genomics Malaria Cell Atlas (Fig. S6A). In doing so, we noted *PlasmoDB-*based life-stage inferences closely followed those inferred from the MCA (Fig. S6B). Consistent with our earlier assessment in *rag1^−/−^* mice (Fig. 5) and the MCA (15), ring and schizont transcriptomes were more uniform compared to trophozoite stages.

We inferred a spectrum of life stages in our data set from youngest rings at the lower left of the UMAP to mature schizonts at the upper right (Fig. 6D; Fig. S5D). Importantly, host inflammation had diverted the transcriptomes of trophozoites. Unsupervised transcriptomic clustering produced 11 clusters, with Clusters 0, 1, and 9 most ring-stage-like, Clusters 8 and 10 most schizont-like, and Clusters 2–7 trophozoite-like (Fig. 6D).

To quantify apparent stage-specific transcriptomic differences, we examined the proportion of transcriptomes in each cluster from each of the host conditions (Fig. 6D; Fig. S5D). While ring- and schizont-like Clusters (0, 1, 8, 9, and 10) had representation from all three conditions, trophozoite Clusters 6 and 7 were almost entirely composed of transcriptomes from naïve mice, while trophozoite Clusters 2, 3, and 5 were dominated by transcriptomes from LPS-conditioning and acute infection (in equal measures) (Fig. 6D). The number of unique mRNA molecules (nUMI) recovered per trophozoite was reduced in pooled Cluster (2 + 3 + 5) compared to Cluster (6 + 7) (Fig. 6E); hence, a trend for systemic inflammation to down-regulate not up-regulate genes (Fig. 6E; Fig. S5E), a phenomenon not observed for rings or schizonts (Fig. S4C). This suggested a quantitative reduction in mRNA content, and thus transcriptional activity, in trophozoites responding to systemic host inflammation. However, qualitative differences were also evident, with 15 genes significantly down-regulated by systemic host inflammation in trophozoites, and only six genes up-regulated (Fig. 6F). The main recurring phenomenon was that 40% of down-regulated genes encoded ribosomal components, suggesting that systemic inflammation had reduced the protein translational capacity of trophozoites (Fig. 6F; Fig. S5F). Consistent with scRNA-seq observation of down-regulation of the parasite transgene eGFP driven off the translation-associated elongation factor 1α promoter (Fig. 6G; Fig. S5G), we noted by flow cytometry at 13 hours post-transfer that translated eGFP protein was reduced in trophozoites by systemic host inflammation, and total RNA content, assessed by Syto84 staining, was also reduced (Fig. 6G; Fig. S5G). Thus, our data revealed a short 4-hour period of *in vivo* exposure to systemic host inflammation reduced transcriptional activity and translational capacity in trophozoite-stage asexual parasites, consistent with our hypothesis that parasites directly sensed and rapidly responded to host inflammation.

## DISCUSSION

Recent studies provide emerging evidence that the maturation rate of blood-stage *Plasmodium* parasites is not constant *in vivo* but can be adjusted, for example, by nutrient starvation or altering circadian rhythms of the host (10, 11). Similarly, we previously reported host-dependent impaired maturation of parasites (12). Here, we extended those findings with evidence that host inflammation altered the plasma environment surrounding parasites *in vivo* and that this altered plasma environment contained inhibitory factors that directly impaired maturation of early trophozoite stages. After only 4 hours of circulation in altered plasma, trophozoites adjusted their transcriptional profiles and protein translational capacity, resulting overall in a lower rate of maturation within RBCs. To the best of our knowledge, this is the first study to demonstrate that the transcriptomes of individual parasites can be modulated by inflammation within a host. This result further emphasizes the dynamic nature of host-parasite interactions in this infection model.

We demonstrated with LPS conditioning that systemic host inflammation alone, in the absence of confounding factors such as ongoing infection (12), slowed the rate at which parasites transited from one generation of RBC to the next. While this is consistent with the idea that host inflammatory responses can impair parasite maturation, other TLR agonists, CpG and Poly I:C, did not elicit such a response. Using serum cytokine data as a proxy for the magnitude of inflammatory response generated, we speculate that the reason only LPS delayed maturation, was an issue of magnitude. Future experiments might examine the effects of larger doses of CpG and Poly I:C, perhaps non-TLR agonists of the innate immune system. Moreover, it is important to note that whether proinflammatory cytokines themselves might have direct anti-parasitic effect on pRBCs remains unresolved, although at least for TNF appears unlikely (32). Rather, the hundreds of other molecules in plasma could also have effects on parasites, as suggested by our metabolomics data.

When we performed *in vitro* cultures with plasma from LPS-conditioned or acutely infected mice, parasite maturation was directly impaired, prior to progression to schizont stages. Given that supplementation with normal plasma did not rescue maturation, we provide causal evidence for inhibitory factors in plasma that directly impaired parasite maturation. This led us to hypothesize possible common inhibitory molecules in the plasma of both acutely infected and LPS-conditioned mice. Untargeted metabolomics permitted an unbiased assessment of the small molecule composition of plasma in which parasites circulated. We observed a perturbation of the plasma metabolome by LPS-conditioning or acute infection compared to naïve mouse plasma, both of which elicited impaired maturation *in vivo*. Metabolomics identified glutarylcarnitine and methylhypoxanthine as elevated in both LPS-conditioned and acutely infected plasma. *Plasmodium* survival depends on host hypoxanthine, inosine, and xanthine for purine synthesis (33). 1-Methylhypoxanthine can bind effectively to and possibly limit the action of hypoxanthine-guanine phosphoribosyl transferase (HGPRTase) (34), an enzyme critical for purine synthesis. Interestingly, hypoxanthine, inosine, and xanthine were also all reduced in the plasma of LPS-conditioned and acutely infected mice supporting the possibility that inhibition of purine synthesis by methylhypoxanthine might have been partly aided by the lack of substrates for this pathway. Interestingly, it was also noted in one of two scRNA-seq experiments that *Plasmodium* HGPRTase was down-regulated in trophozoites after 4 hours of exposure to host inflammation. Taken together, we hypothesize that methylhypoxanthine, in addition to reduced substrate availability and reduced expression of critical parasite enzymes, may have impaired purine synthesis, with subsequent effects on trophozoites. In addition, glutarylcarnitine levels were elevated by LPS conditioning and acute infection. This metabolite is produced from glutaryl-CoA dehydrogenase (GCDH), a mitochondrial enzyme involved in the catabolism of certain amino acids (35). The implications of elevated glutarylcarnitine with respect to parasite maturation remain to be determined. A central assumption in our search for putative inhibitory factors was that a common factor was induced by both LPS conditioning and acute infection. However, it is possible, given the substantial difference in the metabolomic composition of plasma under these two conditions, that different factors and biological processes were responsible for impaired maturation in LPS-conditioned mice compared to acutely infected mice. Further studies will be required to predict additional putative inhibitory factors unique to either condition.

In this study, we hypothesized that metabolomic changes to the host plasma during systemic inflammation could be sensed and responded to by the parasite itself. Transcriptomic methods such as mRNA-sequencing (RNA-seq) offer a system-wide view of unicellular and multi-cellular eukaryotic organisms. In particular, cellular change over a period of hours is captured well using RNA-seq. In our experimental system, *P. berghei* is asynchronous, meaning that all stages of the blood-stage life cycle are present at any given time. The heterogeneity of parasites and the possibility of host mRNA contamination *in vivo* necessitated the adaptation of recent *Plasmodium* scRNA-seq techniques (15). In particular, it was necessary to remove peripheral blood mononuclear cells from our blood samples, achieved by flow cytometric sorting prior to droplet-based scRNA-seq. In addition, while host mRNA was detected in some RBCs, these encoded for hemoglobin almost exclusively and constituted only a small minority of our data. By integrating our *in vivo* data with *in vitro* generated scRNA-seq data held within the MCA (15), we concluded that *in vivo* scRNA-seq analysis of *Plasmodium* parasites was feasible. Interestingly, we noted that as in the MCA, that while rings and schizonts are transcriptomically homogeneous, trophozoites display comparatively higher levels of heterogeneity. It is intriguing to consider what factors might control the differentiation trajectory of parasites as they progress from ring to schizont stages. Whether such transcriptomic variation is functionally relevant remains unclear, although it perhaps indicates that trophozoites more so than other forms are adaptable. We expect future studies will benefit from this capacity to study individual parasites in fine detail.

Importantly, by using scRNA-seq we observed a specific effect of host inflammation, not on rings or schizonts, but on trophozoites. Moreover, we noted specific effects on genes related to protein translation, suggesting a molecular mechanism underpinning delayed maturation of parasites. It was noted that the number of genes differentially expressed as a result of host inflammation was small, although a genome-wide bulk effect on transcription was clearly apparent and was validated by flow cytometry. While our study was designed to detect early changes, assessments at later timepoints might reveal other transcriptional pathways modulated by host inflammation. It should be recognized that although scRNA-seq can detect cellular change over moderate time frames of hours, rapid or even instantaneous cellular response can be undetectable. For example, differential phosphorylation of proteins, protein trafficking, or influx of calcium ions may be biologically crucial and yet would be invisible to scRNA-seq. Future experiments could, for example, explore the phosphoproteome of parasites to provide a fuller picture of how parasites sense and respond to host inflammation.

An attempt to compare the early effect of host inflammation versus antimalarial drug treatment (with rapid acting, sodium artesunate) on parasite transcriptomes was not possible (data not shown), since mRNA recovery was poor after 4 hours of artesunate exposure *in vivo*, perhaps suggestive of rapid killing. This suggests that host inflammation had induced maturation delay, not killing of parasites. In our previous study, profoundly protective parasite-specific antibodies had no effect on the rate of loss of Gen_0_ pRBCs in the presence or absence of LPS-conditioning (5). This indicates two mutually exclusive immune mechanisms could co-exist during acute infection: first, maturation delay induced by altered plasma environments, and second, antibody-mediated blockade of RBC invasion. These mechanisms could be supplemented with anti-malarial drugs to induce rapid killing and/or accelerated parasite removal from circulation (3, 4, 36). Thus, multiple mechanisms exist for controlling parasite numbers *in vivo*.

Finally, we consider broader implications of our findings. First, we propose that host inflammation can reduce parasite population growth via an under-appreciated mechanism, that of reducing parasite maturation rate, not active clearance or killing of parasites in the spleen. The phenomenon of developmentally arrested parasites is not unprecedented, and certain *Plasmodium* species have been reported to enter dormancy (37), either as part of their natural life cycle or as a result of antimalarial drug treatment. Nevertheless, metabolite-mediated inhibition of parasites may constitute a new method for combating parasite growth *in vivo*. Another interpretation of the phenomena presented here is that parasites actively sense an inhospitable host environment and as a result trigger a coping mechanism. We speculate that inflammation-driven alterations to parasite maturation could also contribute to variation between parasites in symptomatic versus asymptomatic infections in endemic regions (38). Our *in vivo* data support a model in which blood-stage *Plasmodium* parasites are aware of their surroundings in the bloodstream and can respond to a highly dynamic host environment. This likely imbues *Plasmodium* parasites with the means to remain adaptable and viable within a mammalian host.

## Data Availability

Mass spectrometry metabolomics raw and extracted feature data have been deposited to the NIH Common Fund's National Metabolomics Data Repository (NMDR) website, the Metabolomics Workbench  (39), where it has been assigned Project IDs PR001195 and PR001670. All scRNA-seq data were uploaded to ArrayExpress with accession number E-MTAB-12954.

## SUPPLEMENTAL MATERIAL

Fig. S1 mbio.01129-23-s0001.tif Experimental repeat of plasma metabolomic and cytokine assessments after LPS conditioning or during acute infection.

Fig. S2 mbio.01129-23-s0002.tif Cytokine analysis of TLR agonists and *Pb*A acute infected mice from untargeted plasma metabolomics experiment.

Fig. S3 mbio.01129-23-s0003.tif Increasing by 4-5-fold the number of CFTR^+^ RBCs transferred into mice does not alter the impaired maturation phenotype.

Fig. S4 mbio.01129-23-s0004.tif Confirmation of impaired maturation during the scRNA-seq experiment.

Fig. S5 mbio.01129-23-s0005.tif Experimental repeat of scRNA-seq assessment of parasites exposed to host inflammation.

Fig. S6 mbio.01129-23-s0006.tif scVI integration of experimental scRNAseq data to the 10x MCA Atlas.

Supplemental legends mbio.01129-23-s0007.docx Legends to Fig. S1 to S6.

ASM does not own the copyrights to Supplemental Material that may be linked to, or accessed through, an article. The authors have granted ASM a non-exclusive, world-wide license to publish the Supplemental Material files. Please contact the corresponding author directly for reuse.
