# Supplementary figures and images for: Systemic host inflammation induces stage-specific transcriptomic modification and slower maturation in malaria parasites
Source: mBio. 2023 Jul 14;14(4):e01129-23. doi: 10.1128/mbio.01129-23 (PMC10470790; doi:10.1128/mbio.01129-23)

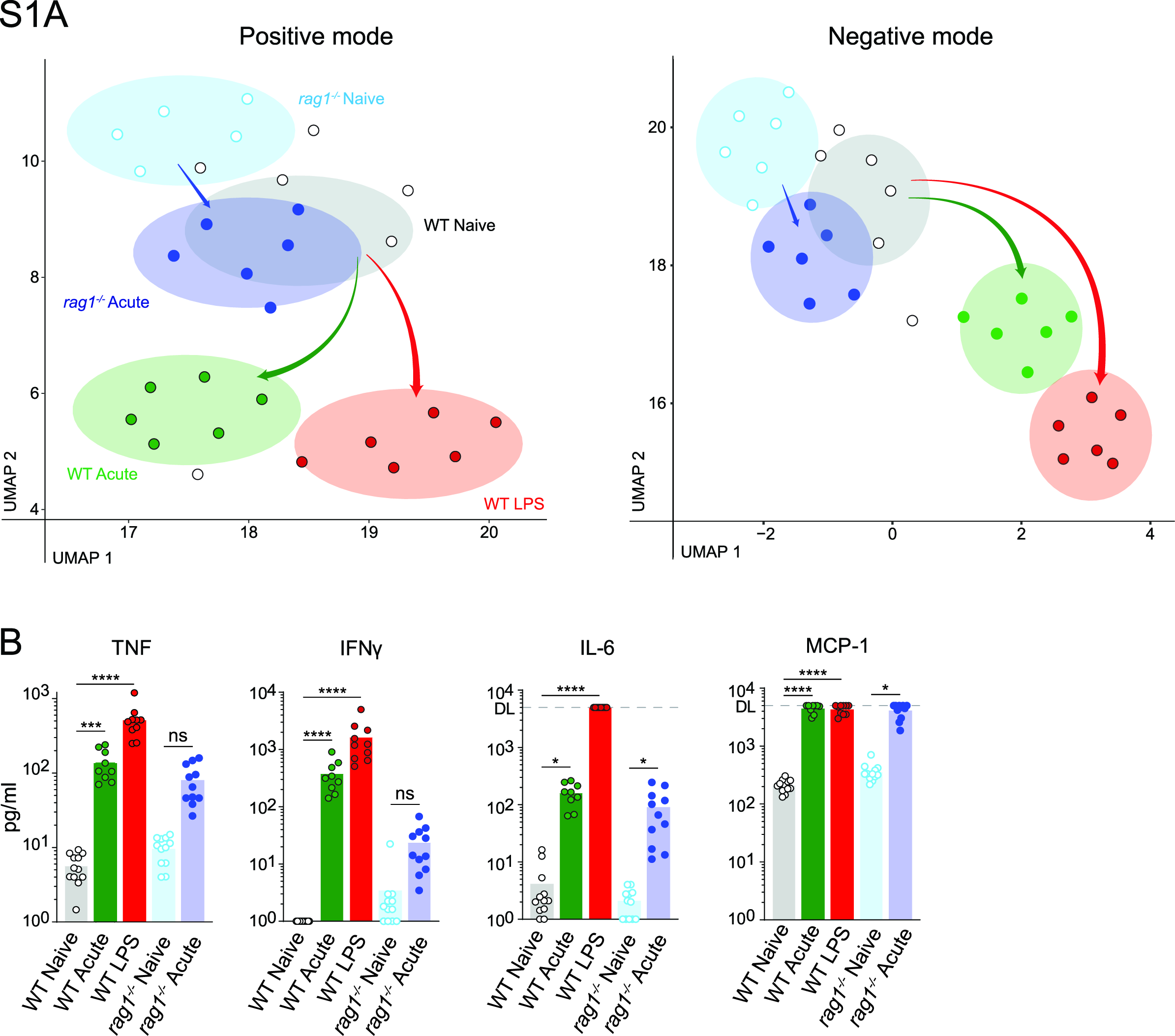

Supplement: Fig. S1 — Experimental repeat of plasma metabolomic and cytokine assessments after LPS conditioning or during acute infection. [file mbio.01129-23-s0001.tif]

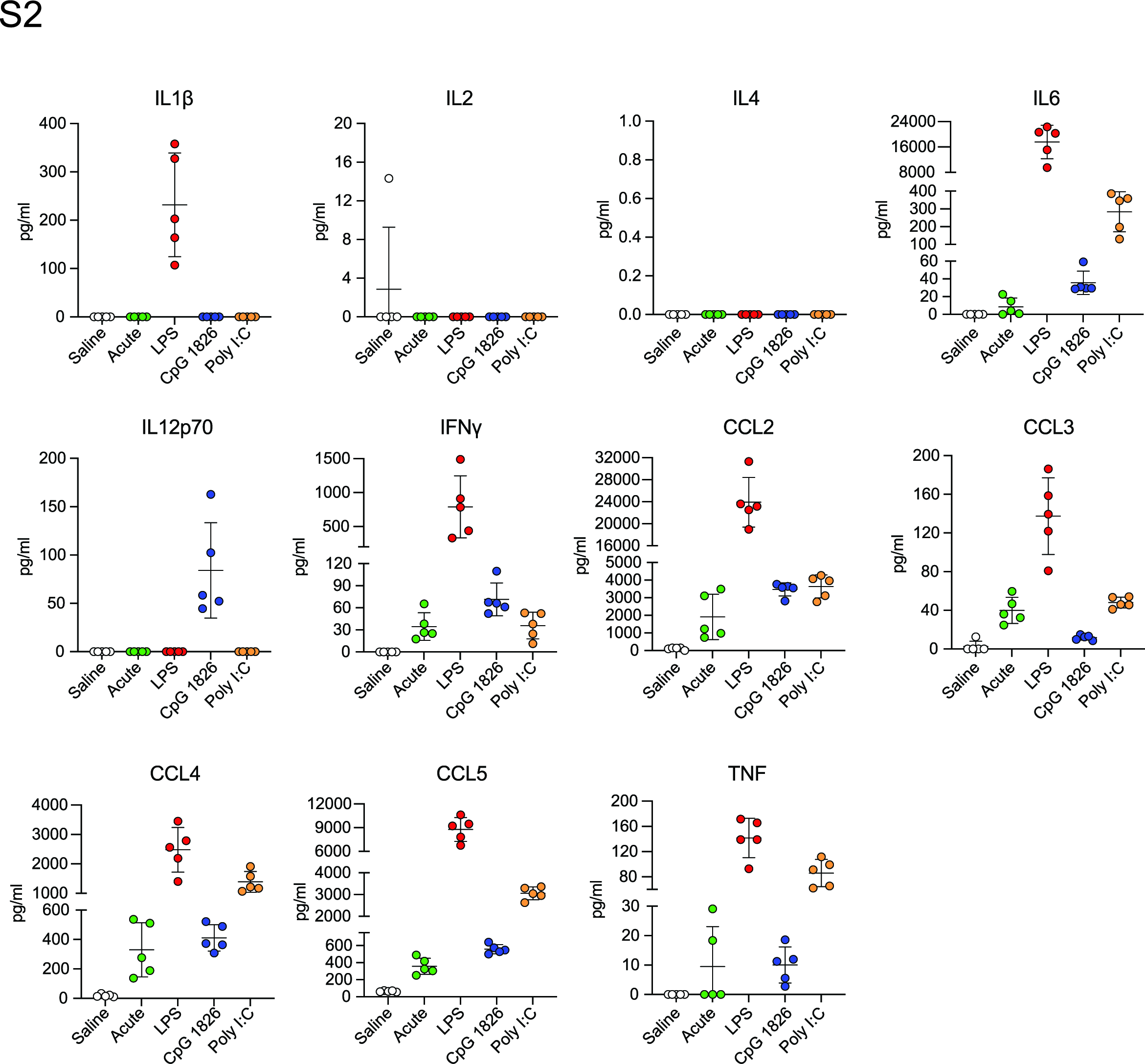

Supplement: Fig. S2 — Cytokine analysis of TLR agonists and PbA acute infected mice from untargeted plasma metabolomics experiment. [file mbio.01129-23-s0002.tif]

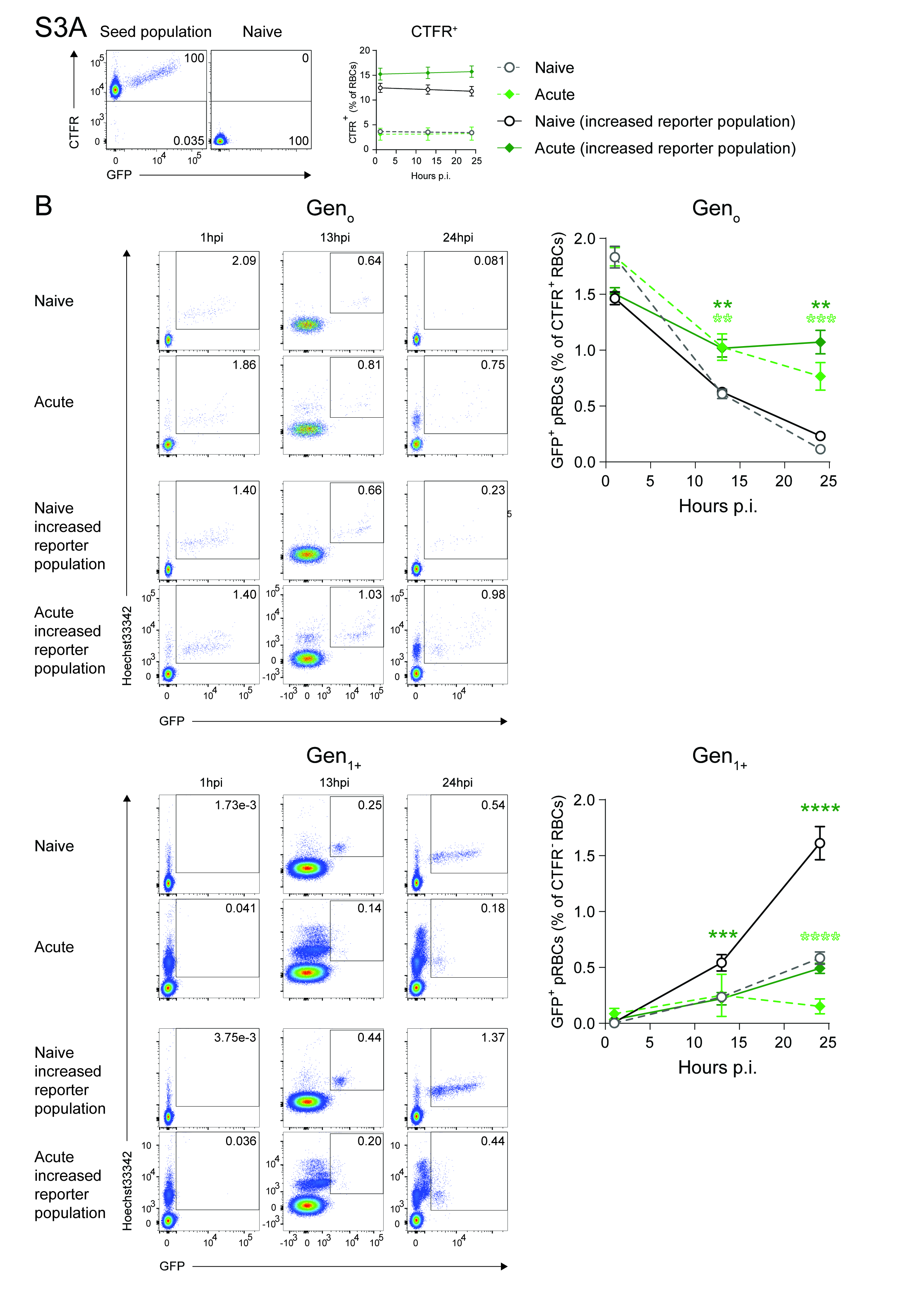

Supplement: Fig. S3 — Increasing by 4-5-fold the number of CFTR+ RBCs transferred into mice does not alter the impaired maturation phenotype. [file mbio.01129-23-s0003.tif]

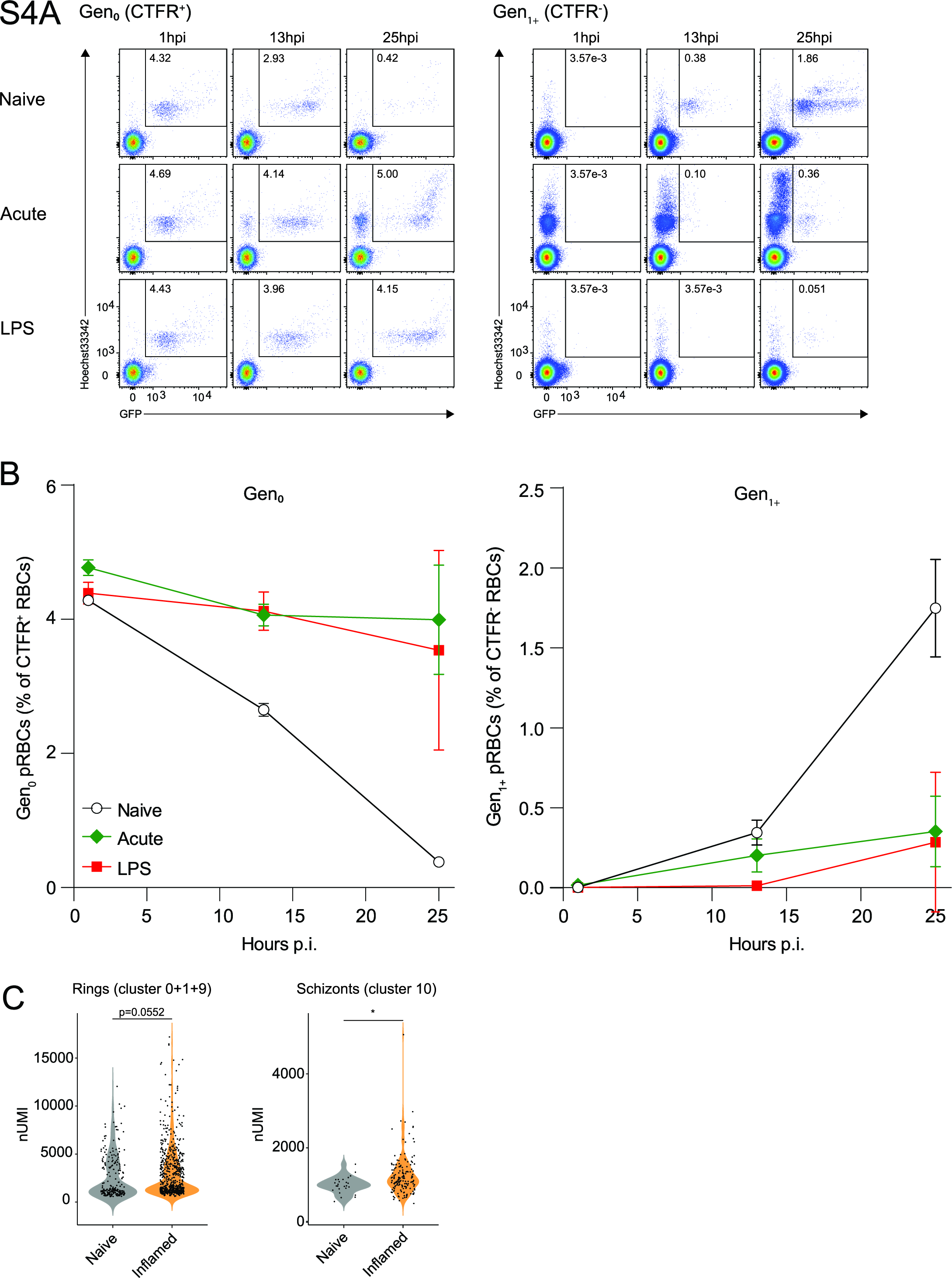

Supplement: Fig. S4 — Confirmation of impaired maturation during the scRNA-seq experiment. [file mbio.01129-23-s0004.tif]

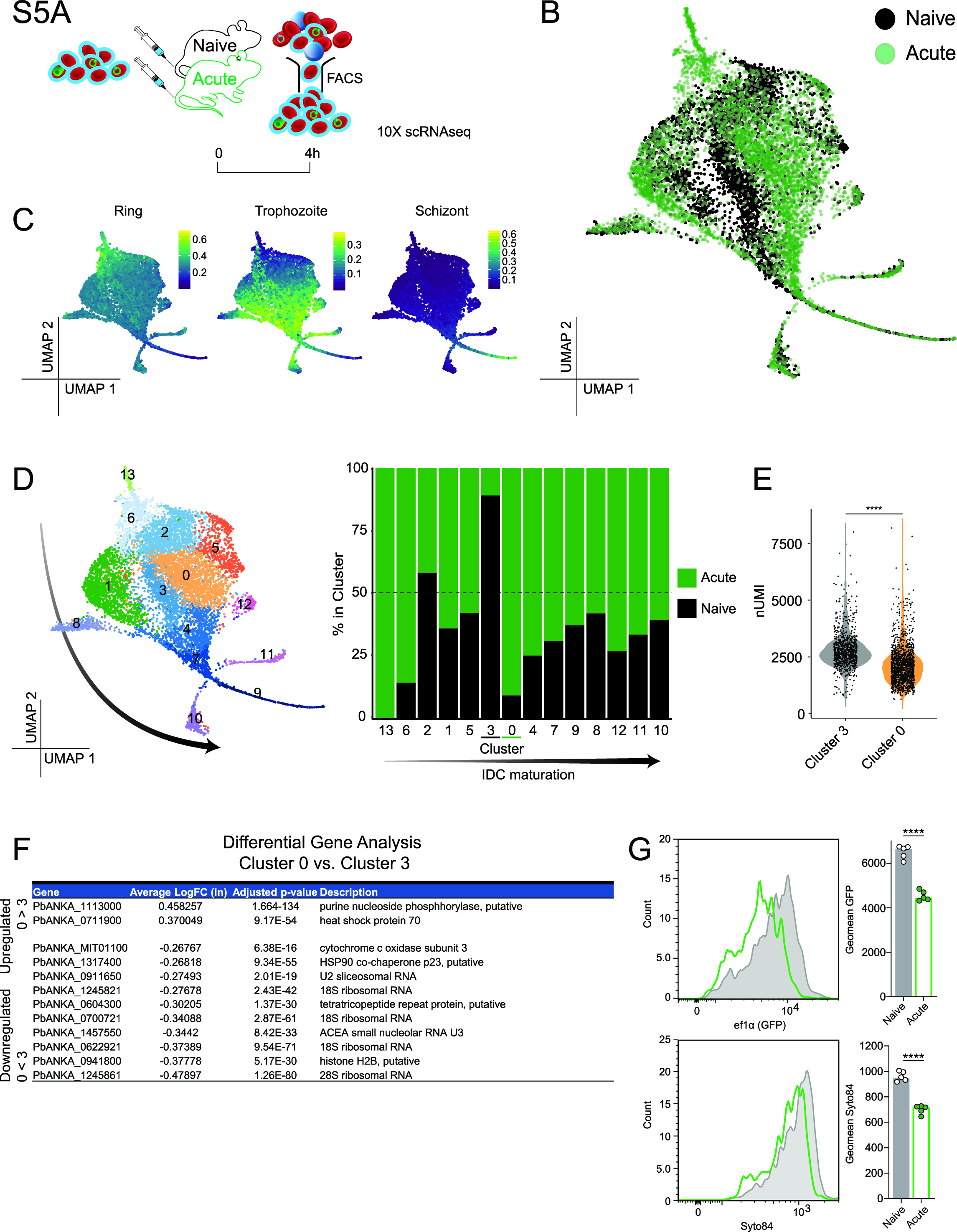

Supplement: Fig. S5 — Experimental repeat of scRNA-seq assessment of parasites exposed to host inflammation. [file mbio.01129-23-s0005.tif]
