# Supplementary material for: Systemic host inflammation induces stage-specific transcriptomic modification and slower maturation in malaria parasites
Source: mBio. 2023 Jul 14;14(4):e01129-23. doi: 10.1128/mbio.01129-23 (PMC10470790; doi:10.1128/mbio.01129-23)
Supplement: Supplemental legends — Legends to Fig. S1 to S6. [file mbio.01129-23-s0007.docx]

**Supplementary Figure Legends**

**Supplementary Figure 1. Experimental repeat of plasma metabolomic and cytokine assessments after LPS conditioning or during acute infection.** A) UMAP dimensionality reduction representation of untargeted LC/MS data obtained for an independent repeat of data in Figure 2, using positive or negative electrospray ionisation (ESI). Dots represent plasma metabolomes from individual mice, shaded ellipses depict centroid and 95% confidence levels for each condition; arrow width indicates Euclidean distance between centroids of groups. B) Plasma cytokine levels in individual mice at time of metabolomic assessment, pooled from two independent repeat experiments (n=5 mice per group per experiment); bars represent mean value (n = 6 mice per group per condition per experiment); dotted line represents the assay detection limit (DL). Data shown is the median with statistical test used non-parametric (Kruskal-Wallis) one-way ANOVA with Dunn’s multiple comparisons test. *p<0.05, ***p<0.001, ****p<0.0001.

**Supplementary Figure 2. Cytokine analysis of TLR agonists and *Pb*ANKA acute infected mice from untargeted plasma metabolomics experiment.** Cytokine Bead Array **(**CBA) analysis of 11 inflammatory markers was performed on plasma from 5-day acute *Pb*ANKA infected mice and mice 9 hours treated with either LPS, PolyI:C, CpG or saline control.

**Supplementary Figure 3. Increasing by 4-5-fold the number of CFTR^+^ RBC transferred into mice does not alter the impaired maturation phenotype.** A) Flow cytometry plots showing CFTR^+^ RBC compared to naïve RBC; graph shows CFTR^+^ RBC dynamics as % of total RBCs for naïve or acutely-infected mice (n=5/group) receiving either the standard number or a 4-5 fold increased number of CFTR^+^ RBC. B) Representative flow cytometry plots of Gen_0_ and Gen_1+_ pRBCs expressed as a percentage of CFTR^+^ RBCt (n=5/group). Statistical significance indicated compared to appropriate naïve control. Statistical analysis: two-way ANOVA with a factor for time-point and for treatment group. Testing for a treatment group effect in Gen_0_ (p<0.0001, F=83.78, df=3) and Gen_1+_ (p<0.0001, F=113.5, df=3). *p<0.05, **p<0.01, ***p<0.001, ****p<0.0001. (Tukey test for multiple comparisons).

**Supplementary Figure 4. Confirmation of impaired maturation during the scRNA-seq experiment.** A) Flow cytometry plots and B) summary graphs of Gen_0_ and Gen_1+_ parasites over time in LPS-conditioned, acutely-infected and control mice (n=5/group). Statistical significance indicated as compared to saline control. C) parasite nUMI per RBC for ring-stages or schizonts in naïve versus pooled acutely-infected and LPS-conditioned groups. Statistical analysis performed mixed-effects analysis for panel B and t=test for C. B) Testing for a treatment group effect in Gen_0_ (p<0.0001, F=55.06) and Gen_1+_ (p<0.0001, ­­F=41.43). C) Testing for a condition effect in rings (p=0.0552, t=3.687, df=1) and schizonts (p=0.0157, t=5.938, df=1). *p<0.05, **p<0.01, ***p<0.001, ****p<0.0001. (Dunnett’s test for multiple comparisons).

**Supplementary Figure 5. Experimental repeat of scRNA-seq assessment of parasites exposed to host inflammation.** A) Schematic showing CFTR^+^ RBC (containing 3.7% *Pb*A-GFP parasites) transferred into acutely-infected or control mice (n=5/group), recovered by cell sorting after 4 hours, and immediately loaded onto a Chromium controller for scRNA-seq analysis. B) 2D-UMAP after PCA of parasite transcriptomes. C) Expression of ring-, trophozoite- and schizont- gene signatures across the UMAP embedding, taken from *PlasmoDB* (<https://plasmodb.org/plasmo/app>). D) Relative contribution of parasites from either group within each of 14 clusters, defined by unsupervised clustering on transcriptomic similarity; arrow indicates inferred rough directionality of maturation from ring to trophozoite to schizonts within the pooled data. E) Number of unique molecular identifiers (nUMI) per cell for cluster 3 and cluster 0. F) Differentially-expressed genes between cluster 0 versus 3 versus ranked by average Log_n_FoldChange. G) Representative histograms of GFP expression driven of the ef1alpha promoter (top) and Syto84 staining (bottom) in non-schizonts 13 hours post-transfer; bar graphs show geometric mean GFP and Syto84 in individual mice (n=5/group). Statistical analyses performed using t-test. E) Testing for a grouped cluster effect (p<0.0001, t=391.9, df=1). G) Testing for a treatment group effect in GFP (p<0.0001, t=11.78, df=8) and Syto84 (p<0.0001, t=11.38, df=8) *p<0.05, **p<0.01, ***p<0.001, ****p<0.0001.

**Supplementary Figure 6. scVI integration of experimental scRNAseq data to the 10X MCA Atlas.** A) scRNAseq datasets from Figure 5, Figure 6 and Supp Figure 5 were integrated with the 10X MCA Atlas using scVI integration to generate an integrated UMAP, coloured by dataset type. B) For each dataset, cluster annotations are labelled and compared to the annotated life stages of the 10X MCA Atlas. Grey dots represent all the cells from the other integrated datasets that form the integrated UMAP. For each integrated dataset, the cluster IDs refer back to the original datasets cluster annotation as shown in Figure 5, Figure 6 and Supp Figure 5.
